# The Association Between “Weekend Warrior” Physical Activity Pattern and Neuropsychological Outcomes: A Systematic Review

**DOI:** 10.3390/bs16050722

**Published:** 2026-05-08

**Authors:** Chen Hong, Jinglei Zhao, Jinrui Xue, Sicheng Liu, Shiyu Wang, Wenrui Zhao, Boyi Zong, Xiaoyou Zhang

**Affiliations:** 1 School of Physical Education, Hubei University, Wuhan 430062, China; 202421109011964@stu.hubu.edu.cn (C.H.); 202521109012004@stu.hubu.edu.cn (J.Z.); 202521109012014@stu.hubu.edu.cn (J.X.); 202421109011992@stu.hubu.edu.cn (S.L.); 2 College of Physical Education and Health, East China Normal University, Shanghai 200241, China; 52261000010@stu.ecnu.edu.cn; 3 Key Laboratory of Adolescent Health Assessment and Exercise Intervention of Ministry of Education, East China Normal University, Shanghai 200241, China; 4 College of Physical Education and Health Sciences, Zhejiang Normal University, Jinhua 321004, China; zhaowenrui108@zjnu.edu.cn; 5 School of Sport Sciences, Nanjing Normal University, Nanjing 210023, China; boyi0303@126.com

**Keywords:** physical activity, weekend warrior, neuropsychological outcomes, neurodegenerative diseases, mental health, cognitive function

## Abstract

Physical inactivity among adults is a major contributor to the escalating global burden of neurodegenerative diseases and mental disorders. The “Weekend Warrior” (WW) pattern—characterized by condensing the recommended volume of physical activity (PA) into one or two days—has emerged as a potential strategy for time-constrained adults. However, a systematic synthesis comparing the potential association of the WW pattern on neuropsychological outcomes (e.g., neurodegenerative diseases, mental health conditions and cognitive function) with those of inactive (IA) and regular exercise (RE) patterns remains limited. This systematic review aimed to assess the associations of the WW pattern with neurodegenerative diseases (e.g., dementia, Parkinson’s disease), mental health conditions (e.g., depression, anxiety, psychological distress), and cognitive function (CF), and to compare these neuropsychological outcomes with those associated with IA and RE patterns. A systematic search of PubMed, Web of Science, ScienceDirect, and Chinese databases (CNKI, Wanfang) was conducted for observational studies published from inception to October 2025. Thirteen studies (5 cohort and 8 cross-sectional studies) involving adults were included. The assessment of methodological quality was conducted using the Agency for Healthcare Research and Quality (AHRQ) methodology and the Newcastle–Ottawa Scale (NOS). The majority of included studies indicated that the WW pattern was inversely associated with the risks of neurodegenerative diseases and mental health conditions and enhanced CF, exhibiting associations of a similar magnitude to those of RE. Furthermore, the synthesis highlighted prevailing gaps in the extant literature, particularly regarding the lack of randomized controlled trials, insufficient control for confounding variables such as social context, and the limited investigation into the neurobiological mechanisms underlying these associations. This systematic review emphasizes the WW pattern as a viable and time-efficient strategy that promotes positive neuropsychological outcomes, exhibiting associations of a similar magnitude to those observed with RE. The findings substantiate the flexibility inherent in PA guidelines, underscoring that adhering to recommended PA volumes is highly beneficial, even when accumulated within a condensed timeframe. Future research should prioritize randomized controlled trials and investigations into neurobiological mechanisms to further validate causal relationships and elucidate the underlying mechanisms driving these associations.

## 1. Introduction

Physical activity (PA) confers significant benefits in the prevention and alleviation of diverse neurodegenerative diseases (e.g., dementia and Parkinson’s disease; Liu et al., 2019) and mental health conditions (e.g., depression, anxiety, and psychological distress; American Psychiatric Association, 2022), while also contributing to the enhancement of cognitive function (CF) (Harvey, 2019). Within the context of this review, we collectively conceptualize these three domains—neurodegenerative diseases, mental health conditions, and CF—under the broad umbrella of neuropsychological outcomes to comprehensively capture the full spectrum of neuropsychological health. Crucially, these neuropsychological outcomes are intrinsically interconnected: mental health conditions frequently exacerbate cognitive deterioration, which in turn serves as a core clinical manifestation and a primary precursor to neurodegenerative pathologies (Rashidi-Ranjbar et al., 2020). Extensive research demonstrates that individuals who engage in regular exercise (RE) have a reduced risk of developing dementia and Parkinson’s disease, experiencing adverse psychological states, and suffering from cognitive decline (Fang et al., 2018; Hooker et al., 2022; Iso-Markku et al., 2022; Singh et al., 2025). Despite widespread recognition of the critical role exercise plays in maintaining neuropsychological health, approximately 27.5% of adults worldwide do not meet the PA levels recommended by the World Health Organization (WHO) (Guthold et al., 2018). Should physical inactivity persist, it is projected that nearly 499 million new cases of non-communicable diseases, including depression and anxiety, will emerge by 2030 (Santos et al., 2023). Concurrently, driven by factors such as population aging, the incidence and prevalence of neurodegenerative diseases are steadily rising, with case numbers anticipated to surge over the coming decades. This trend presents a substantial challenge to global public health systems (Hao & Chen, 2025; L. Xu et al., 2025).

The current World Health Organization (WHO) *Guidelines on PA and sedentary behaviour* recommend that adults engage in 150 to 300 min of moderate-intensity or 75 to 150 min of vigorous-intensity PA per week to derive health benefits (WHO, 2020). However, daily workloads, familial responsibilities, and other social obligations often hinder busy demographics, such as “white-collar” workers, from maintaining a routine of daily PA. In response to this constraint, the “Weekend Warrior” (WW) pattern—originally proposed by Lee et al. (2004) and characterized by achieving 150 min of moderate-to-vigorous physical activity (MVPA) per week, with at least 50% of the total volume accumulated within 1–2 days—has garnered renewed scientific interest (Kunutsor et al., 2023; Min et al., 2024) for its potential to elicit benefits comparable to RE through concentrated, condensed bouts of activity.

Existing research typically employs current guideline thresholds to stratify individuals into three distinct PA patterns: Inactive (IA) (<150 min/week of MVPA), RE (≥150 min per week of MVPA, but not meeting WW criteria), and WW (≥150 min per week of MVPA, with at least 50% of the total volume accumulated within 1–2 days) (Khurshid et al., 2023; Mahe et al., 2024; Min et al., 2024; O’Donovan et al., 2024). PA has been extensively substantiated as a cost-effective, non-pharmacological intervention for ameliorating various neurodegenerative diseases and mental health conditions as well as enhancing CF (Conn, 2010; Mammen & Faulkner, 2013; Rebar et al., 2015; Singh et al., 2025; Wipfli et al., 2008). However, the majority of existing literature focuses on traditional RE, leaving a scarcity of evidence regarding the WW pattern, which shows inconsistent associations with neuropsychological outcomes. For instance, while R. Chen et al. (2023) and S. Xu et al. (2025) discovered the WW pattern lowers depression risk comparably to RE, the cross-sectional evidence from a Brazilian population indicates no notable improvement in depressive symptoms (de Victo et al., 2025). This inconsistency obscures a definitive understanding of whether the WW pattern is truly effective for neuropsychological outcomes and whether its benefits align with those of RE pattern. Given that the WW pattern represents a vital potential strategy for time-poor populations to maintain health, clarifying these controversies is paramount for informing public health guidelines. Crucially, the current literature lacks sufficient critical context regarding these inconsistencies, which likely stem from highly heterogeneous operational definitions of the WW pattern across studies and variations in PA measurement tools (e.g., objective accelerometry versus subjective self-reports). Furthermore, a distinct research gap persists: the vast majority of existing evidence relies on indirect comparisons against inactive baselines, lacking direct, head-to-head statistical comparisons between the WW and RE patterns.

Building on this evidence, the primary objective of this systematic review is to explicitly evaluate the associations between the WW pattern and neuropsychological outcomes (neurodegenerative diseases, mental health conditions, and cognitive function), and to assess whether the magnitude of these associations is similar to that observed with regular exercise (RE). Specifically, we aim to address the following three core questions:

(1) Relative to the IA, does the WW pattern show a potential association with outcomes comparable to those of RE, thereby being associated with a lower risk of neurodegenerative diseases (e.g., dementia and Parkinson’s disease)? (2) Compared to IA, is the WW pattern associated with a lower risk of prevalent mental health conditions (e.g., depression, anxiety and psychological distress), and how does this association compare to that of RE? and (3) How does the WW pattern influence CF relative to both the IA and RE?

## 2. Method

### 2.1. Search Strategy

The current study adhered strictly to the protocols outlined in the Preferred Reporting Items for Systematic Reviews and Meta-Analyses (PRISMA) statement. A comprehensive literature search was performed across PubMed, Web of Science, ScienceDirect, and Chinese databases, including the China National Knowledge Infrastructure (CNKI) and Wanfang Data. The screening window encompassed all available literature from the inception of these databases through the exact date of the last search on 25 October 2025. No controlled vocabulary terms (e.g., MeSH) were utilized; instead, search terms were combined in the following manner: (“weekend warrior” OR “weekend warriors” OR “WW”) AND (“neurodegenerative diseases” OR “dementia” OR “Parkinson’s disease” OR “mental health” OR “mental disorder” OR “depression” OR “anxiety” OR “psychological distress” OR “cognitive function”). The search was conducted without language restrictions. Notably, within the ScienceDirect database, we restricted the screening keyword solely to “weekend warrior”. In addition, secondary searches were performed for other sources, including the reference lists and citation indices of the pre-selected studies, to identify eligible articles that the electronic database search might have missed. The protocol for this review was not previously registered.

The search strategy, study selection, data extraction, and quality assessment were done independently by two authors (C.H. and J.Z.), following a standardized procedure. Disagreements were resolved by consensus or by consulting the third author (J.X.).

### 2.2. Eligibility Criteria

#### 2.2.1. Inclusion Criteria

The final selection comprised 13 empirical studies written in English and sourced from peer-reviewed journals, comprising both cross-sectional and cohort studies. The included study populations consisted of adults, with no restrictions regarding gender or geographic region. All included studies utilized PA levels measured via accelerometers or self-reports as the primary basis for classification, categorizing participants strictly into WW, RE, or IA groups. These studies investigated the effects of the WW pattern in comparison to RE modes.

#### 2.2.2. Exclusion Criteria

The exclusion criteria were as follows: (1) articles that failed to differentiate the WW pattern from other PA patterns (e.g., RE); (2) studies in which the WW pattern was not defined as the primary exposure variable; (3) review articles and conference proceedings; (4) research lacking relevant outcome measures concerning neurodegenerative diseases, mental health conditions, or CF; (5) studies focusing on physical diseases or involving prescription pharmaceutical interventions; and (6) animal studies.

### 2.3. Data Extraction

The following data were extracted from the included studies: (1) first author, year of publication, and country; (2) source of data; (3) methodology utilized for assessing PA; (4) sample size and baseline characteristics of participants; (5) study characteristics (including design and duration); (6) definitions employed for PA patterns (WW, RE, and IA); and (7) results and key findings directly pertaining to the dependent variables.

The first and second authors (C.H. and J.Z.) independently extracted data regarding the primary indicators, while the third author (J.X.) verified the accuracy and completeness of the extracted dataset. Additionally, random cross-checks were performed to mitigate the risk of data extraction errors. Any differences were resolved by consensus.

### 2.4. Data Synthesis

Given the substantial heterogeneity among included studies regarding PA definitions, outcome measures, statistical models, and data sources, a quantitative meta-analysis was deemed inappropriate. The rationale is threefold: First, concerns regarding population independence arose, as multiple studies analyzed overlapping cohorts from large databases such as the UK Biobank and NHANES, creating a risk of double-counting and estimation bias if statistically pooled. Second, discrepancies existed in the definition and measurement of the WW exposure; studies utilized varying thresholds to define the WW pattern and employed inconsistent assessment methodologies, with the mixing of objective accelerometry and subjective self-reports introducing further conceptual and methodological variability. Finally, the outcomes and their corresponding effect measures were incongruent, as cohort studies reported hazard ratios (HRs) for disease incidence based on differing case definitions and covariate adjustments, cross-sectional studies reported odds ratios (ORs) for neuropsychological outcomes, while studies evaluating CF primarily reported beta coefficients (β) for continuous performance scores, thereby precluding direct quantitative synthesis. Consequently, a narrative synthesis approach was adopted to summarize and compare findings across studies. The analysis was performed based on the following criteria: type of outcome (e.g., neurodegenerative diseases, mental health conditions, CF), direction and magnitude of associations, and consistency of results across diverse study designs and populations.

### 2.5. Study Quality Assessment

To appraise the methodological rigor of cohort studies, we utilized the Newcastle–Ottawa Scale (NOS), which scrutinizes three key domains: selection, comparability, and outcome assessment. The scale assigns a star rating from 0 to 9, where a higher count indicates greater study robustness. We stratified quality into three tiers: low (0–3 stars), moderate (4–6 stars), and high (7–9 stars). For cross-sectional studies, assessment was performed using the 11-item checklist developed by the Agency for Healthcare Research and Quality (AHRQ), where items answering “Yes” were awarded 1 point, while “No” or “Unclear” responses received 0. Based on cumulative scores, study quality was categorized as low (0–3), moderate (4–7), or high (8–11). Two reviewers (C.H. and J.Z.) independently carried out all quality assessments, whereby instances of discordance were reconciled through dialogue or by seeking arbitration from a third reviewer (J.X.) to ensure consensus.

## 3. Results

### 3.1. Study Selection

**Figure 1:**
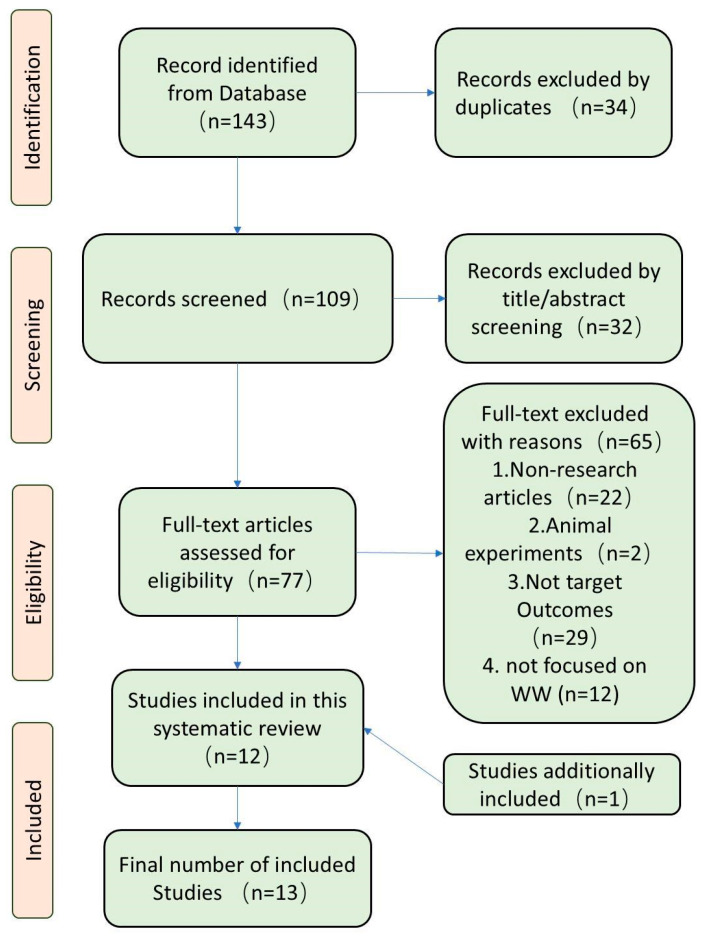
Flowchart of the study selection process for the systematic review.

Figure 1 presents a flowchart summarizing the study selection process and results recommended by the PRISMA guidelines. A total of 143 potentially relevant records were identified from databases and additional sources, including the reference lists of review articles and original papers. Following the exclusion of duplicates, the screening of titles and abstracts, and the exclusion of ineligible literature by assessing full-text articles, 13 studies (R. Chen et al., 2023; Z. Chen et al., 2025; de Victo et al., 2025; Hamer et al., 2017; Liang et al., 2023; Liu et al., 2025; Min et al., 2024; Ning et al., 2024, 2025; O’Donovan et al., 2025; Wu et al., 2024a, 2024b; S. Xu et al., 2025) involving over 100,000 participants were included for systematic review.

### 3.2. Characteristics of Included Studies

The basic characteristics of the included studies are summarized in Table 1. A total of 13 human studies were incorporated, comprising 5 cohort studies (Liu et al., 2025; Min et al., 2024; Ning et al., 2024, 2025; O’Donovan et al., 2025) and 8 cross-sectional studies (R. Chen et al., 2023; Z. Chen et al., 2025; de Victo et al., 2025; Hamer et al., 2017; Liang et al., 2023; Wu et al., 2024a, 2024b; S. Xu et al., 2025). Sample sizes varied across studies, ranging from 2877 to 108,011 participants. Data were derived from multiple sources: six studies (R. Chen et al., 2023; Z. Chen et al., 2025; Liang et al., 2023; Wu et al., 2024a, 2024b; S. Xu et al., 2025) utilized the National Health and Nutrition Examination Survey (NHANES) (United States), four (Liu et al., 2025; Min et al., 2024; Ning et al., 2024, 2025) were based on the UK Biobank (United Kingdom), one (Hamer et al., 2017) employed pooled data from the Health Survey for England (HSE) and the Scottish Health Survey (SHS) (United Kingdom), one (de Victo et al., 2025) was conducted using data from a health screening program at the Preventive Medicine Center of Hospital Israelita Albert Einstein (Brazil), and another (O’Donovan et al., 2025) in Mexico City Prospective Study (Mexico).

All studies enrolled adults aged 18 years or older, with a primary focus on middle-aged and older populations. The gender distribution across the included studies was generally balanced. PA was assessed primarily through accelerometry and self-report methods. With the exception of a single study published in 2017 (Hamer et al., 2017), the majority of the studies were published during the period from 2023 to 2025. Specifically, two studies were published in 2023 (R. Chen et al., 2023; Liang et al., 2023), four in 2024 (Min et al., 2024; Ning et al., 2024; Wu et al., 2024a, 2024b), and six in 2025 (Z. Chen et al., 2025; de Victo et al., 2025; Liu et al., 2025; Ning et al., 2025; O’Donovan et al., 2025; S. Xu et al., 2025).

For cohort studies, the follow-up duration spanned from 6.3 to 16.2 years. Studies examined the associations between PA patterns—particularly the WW pattern—and a range of neuropsychological outcomes, including Parkinson’s disease, dementia, depression, and anxiety. Relevant symptom assessment scales and outcome measures are detailed in Table 1, while Table 2 specifies the exercise characteristics of the WW programs. Furthermore, given the inconsistencies in classification criteria across studies, Table 3 details the diverse operational definitions of the WW pattern.

### 3.3. Quality Assessment

Detailed results of the quality assessments for cohort and cross-sectional studies are presented in Table 4 and Table 5, respectively. The five cohort studies (Liu et al., 2025; Min et al., 2024; Ning et al., 2024, 2025; O’Donovan et al., 2025) achieved Newcastle-Ottawa Scale (NOS) scores ranging from 8 to 9, classifying them all as high-quality research. All eight cross-sectional studies (R. Chen et al., 2023; Z. Chen et al., 2025; de Victo et al., 2025; Hamer et al., 2017; Liang et al., 2023; Wu et al., 2024a, 2024b; S. Xu et al., 2025) attained Agency for Healthcare Research and Quality (AHRQ) scores exceeding 10, indicating robust overall methodological quality. The vast majority of studies clearly defined their study populations and assessment methodologies, with the primary potential limitation being the inherent challenge of causal inference associated with cross-sectional designs. Key strengths of these studies include explicit population selection criteria and substantial inter-group comparability. A minor limitation regarding follow-up completeness was noted, specifically the lack of explicit reporting on attrition rates and descriptions of participants lost to follow-up. On aggregate, the eligible studies demonstrated a high degree of methodological robustness. However, we must emphasize that high methodological scores do not equate to a high strength of evidence. The overall strength of evidence in this review is downgraded by significant measurement bias. Because most included studies relied on self-reported measures for PA rather than objective tools (e.g., accelerometers), the validity of the primary exposure is subject to recall and social desirability biases. Therefore, despite the methodological soundness of the study designs, the current evidence base remains somewhat limited in strength.

### 3.4. Neurodegenerative Diseases

A total of 4 out of the 13 quantitative studies (Min et al., 2024; Ning et al., 2024, 2025; O’Donovan et al., 2025) examined the association of the WW pattern with neurodegenerative diseases (specifically dementia and Parkinson’s disease). Regarding PA assessment, two of these studies utilized objective accelerometry (Min et al., 2024; Ning et al., 2024), while the remaining two relied on self-reported measures (Ning et al., 2025; O’Donovan et al., 2025). All four employed a prospective cohort design, with mean follow-up durations ranging from 7.93 to 16.2 years. It is important to note, however, that the comparability between the WW and RE patterns in these cohort studies was inferred indirectly. Specifically, both patterns were exclusively compared against the IA baseline to derive their respective effect sizes (e.g., HRs), rather than being subjected to direct head-to-head statistical testing.

#### 3.4.1. Dementia

All four studies (Min et al., 2024; Ning et al., 2024, 2025; O’Donovan et al., 2025) reported on the association of the WW pattern with dementia. The findings consistently revealed that, relative to the inactive (IA) group, the WW group demonstrated a protective association against dementia, exhibiting risk-reduction benefits comparable to those of the RE group. Notably, only the study conducted in the Mexican population defined mild dementia as an MMSE ≤ 22. It reported that the WW pattern conferred a protective benefit against mild dementia (HR = 0.75, 95% CI [0.61, 0.91]), comparable to that of RE (HR = 0.84, 95% CI [0.75, 0.95]).

Subgroup and interaction analyses provided further nuanced insights. However, only one study (Ning et al., 2025) investigated the interaction between PA patterns and sedentary behavior, revealing that the connection between the WW pattern and a lower risk of dementia remained significant within the high sedentary time subgroup (daily average ≥ 8.52 h; HR = 0.69, 95% CI [0.54, 0.87]), whereas RE demonstrated no significant association in this high-sedentary group (HR = 0.86, 95% CI [0.66, 1.06]).

Furthermore, a potential dose-dependent limit was observed: another study by the same author (Ning et al., 2024) indicated that when the WW pattern was defined as concentrating 50% of the total PA volume over 1–2 days (regardless of continuity), the pattern robustly lowered dementia risk even when the weekly MVPA threshold was increased from 150 to 300 min. Conversely, when the definition was restricted to concentrating more than 75% of the activity volume within 1–2 consecutive days (as opposed to simply over 1–2 days), this association was no longer statistically significant beyond the 300 min threshold (HR = 0.59, 95% CI [0.19, 1.85]).

#### 3.4.2. Parkinson’s Disease

Two studies (Min et al., 2024; Ning et al., 2024) reported that the WW and RE patterns were linked to a decreased risk of Parkinson’s disease, conferring comparable benefits.

In the Subgroup analyses, Min et al. (2024) notably observed that this trend remained consistent across sexes, with similar risk reductions observed in both men and women. However, echoing the findings for dementia, Ning et al. (2024) demonstrated that when the MVPA threshold was elevated to 300 min and the definition strictly required accumulating the vast majority (>75%) of the volume within 1–2 consecutive days, the connection between the WW pattern and a lower risk of Parkinson’s disease ceased to maintain statistical significance (HR = 0.27, 95% CI [0.04, 1.95]).

### 3.5. Mental Health Conditions

#### 3.5.1. Depression

Out of the 13 articles included, five investigated the correlation between the WW pattern and depression. These comprised one cohort study (Liu et al., 2025) employing objective accelerometry to measure PA and four cross-sectional studies (R. Chen et al., 2023; de Victo et al., 2025; Liang et al., 2023; S. Xu et al., 2025) relying on self-reported questionnaires. The majority of the evidence indicates that, compared to IA, the WW pattern significantly lowers the risk of depression, delivering protective benefits comparable to those of the RE pattern. Notably, regarding this comparability, only R. Chen et al. (2023) conducted a direct statistical comparison by utilizing the RE pattern as the reference baseline. However, one cross-sectional study (de Victo et al., 2025) reported inconsistent findings, showing no significant association for the WW pattern.

The only cohort study (Liu et al., 2025) reported no discernible difference in depression risk between the WW and RE groups. Furthermore, compared to the IA population, individuals adopting the WW pattern exhibited a marked reduction in depression risk (HR = 0.72, 95% CI [0.65, 0.80]), a protective benefit consistent with that observed in the RE mode (HR = 0.74, 95% CI [0.66, 0.84]).

Furthermore, subgroup and interaction analyses provided critical insights, particularly regarding sedentary populations. Liu et al. (2025) investigated the association between different exercise patterns and depression risk across varying stratifications of sedentary time (≤6, 7–12, ≥13 h/day) and light PA (≤60, 61–150, ≥151 min/day); results indicated that the WW pattern yielded the most pronounced risk reduction (HR = 0.55, 95% CI [0.34, 0.90]) among individuals with high sedentary time (≥13 h/day) and low light PA (≤60 min/day). Furthermore, stratified analysis by genetic risk profile revealed that across all genetic risk categories (low, intermediate, and high), both the RE and WW groups demonstrated a consistent trend of attenuated susceptibility to depression (Liu et al., 2025).

Among the remaining cross-sectional studies (R. Chen et al., 2023; S. Xu et al., 2025; de Victo et al., 2025), subgroup analyses were also conducted specifically focusing on sedentary populations. Uniquely, R. Chen et al. (2023) distinguished itself as the only investigation to conduct a direct head-to-head statistical comparison between the WW and RE patterns. Their findings confirmed that while the WW group showed a numerically greater risk reduction (OR = 0.43, 95% CI [0.25, 0.74]) than the RE group (OR = 0.46, 95% CI [0.39, 0.54]) compared to inactivity, this direct comparison revealed no statistical significance (OR = 0.95, 95% CI [0.54, 1.68]), thereby providing the sole direct evidence that the WW pattern matches the efficacy of RE. This finding aligns with the consensus supported by Liang et al. (2023), Liu et al. (2025) and S. Xu et al. (2025), confirming that, compared to inactivity, the WW pattern significantly lowers depression risk, delivering benefits comparable to those of RE.

However, a notable divergence was observed in this cross-sectional study conducted in Brazil (de Victo et al., 2025). The evidence demonstrated that, following adjustment for covariates such as age, gender, and sedentary behavior, only the RE pattern was significantly associated with a lower risk of depression (OR = 0.68, 95% CI [0.62, 0.75]), whereas no significant association was observed for the WW pattern (OR = 0.96, 95% CI [0.74, 1.25]).

#### 3.5.2. Anxiety

Two investigations, both relying on indirect comparisons, specifically assessed the correlation linking the WW pattern to anxiety outcomes: a cohort study utilizing objective accelerometry (Liu et al., 2025) and a cross-sectional study based on self-reported data (Z. Chen et al., 2025). Both studies found that, compared to the IA group, the WW group reported a lower risk of anxiety, demonstrating a potential association similar to that observed in the RE group (Z. Chen et al., 2025; Liu et al., 2025).

The sole cohort study (Liu et al., 2025) also evaluated the interactive effects of PA patterns and Polygenic Risk Scores (PRS) on anxiety risk. Results demonstrated that across all genetic risk categories, both the RE and WW groups exhibited comparable reductions in anxiety risk. Notably, the beneficial effects of the WW pattern were particularly pronounced among individuals characterized by high sedentary behavior and insufficient light PA. Individuals with a low PRS who actively engaged in MVPA—regardless of whether they followed the RE or WW pattern—exhibited the lowest risk of anxiety (Liu et al., 2025). In the interaction analysis regarding sedentary time and light PA, the study indicated that the WW pattern yielded the most optimal reduction in anxiety risk (HR = 0.62, 95% CI [0.40, 0.95]) among participants with sedentary time ≥ 13 h/day and light PA ≤ 60 min/day (Liu et al., 2025).

The cross-sectional study by Z. Chen et al. (2025) indicated that, compared to IA, both the WW pattern and RE exhibited a lower risk of anxiety. Furthermore, based on indirect comparisons of their respective effect sizes against the IA baseline, the protective benefits of the WW pattern appeared numerically comparable to those of the RE mode. However, the impact of the WW pattern was not statistically significant among high-income individuals (OR = 0.93, 95% CI [0.60, 1.43]), whereas the association was particularly pronounced among low-income populations (OR = 0.52, 95% CI [0.33, 0.83]) and patients with diabetes (OR = 0.29, 95% CI [0.11, 0.74]) (Z. Chen et al., 2025).

#### 3.5.3. Psychological Distress

Among the 13 selected studies, a single cross-sectional study (Hamer et al., 2017) utilizing self-reported PA data did not focus on specific psychiatric diagnoses but rather utilized psychological distress (defined as a GHQ-12 score > 3) as the dependent variable. This study investigated the comparative effectiveness of the WW pattern and RE in ameliorating psychological distress among adults through an indirect comparison. The findings supported an association between PA dose and mental health (Hamer et al., 2017), with engagement in the WW pattern being specifically associated with a reduction in psychological distress (OR = 0.68, 95% CI [0.63, 0.73]).

### 3.6. Cognitive Function

Two cross-sectional studies (Wu et al., 2024a, 2024b) evaluated the association between the WW pattern and CF. It is important to note that both of these studies relied exclusively on self-reported PA measures and indirect comparisons. Overall, the evidence suggests that the WW pattern effectively enhances various cognitive domains compared to IA. However, the comparability of its associated outcomes to those of RE appears to be highly contingent upon specific demographic and psychological characteristics, notably gender and the presence of depressive symptoms.

Both studies utilized a standardized neuropsychological test battery as the outcome measure, comprising the CERAD-WL for assessing memory function, the Animal Fluency (AF) test for verbal fluency, and the Digit Symbol Substitution Test (DSST) for evaluating processing speed and executive function.

Stratified analyses revealed distinct cognitive responses to the WW pattern across different subpopulations. One study (Wu et al., 2024a) assessed depressive symptoms using the Patient Health Questionnaire-9 (PHQ-9) (Manea et al., 2015) to stratify the analysis. The results indicated a significant positive correlation between PA patterns (and duration) and improvements in CF. Compared to non-depressed individuals, an increase of 1 h per week in PA duration (whether via the WW or RE pattern) demonstrated a significant positive association with the amelioration of CF within the cohort suffering from depression (Wu et al., 2024a). Among older adults with depressive symptoms, only RE exhibited a distinct positive linkage with cognitive gains (β = 0.15, 95% CI [0.04, 0.25]), whereas the association between the WW pattern and the outcome did not reach statistical significance in this subgroup (β = 0.22, 95% CI [−0.02, 0.47]). Additionally, smoothing curve fitting within the study revealed that the inflection point for exercise benefits was 0.92 h per week for the non-depressed group and 0.58 h per week for the depressed group.

Conversely, findings from the specific cohort targeting older women revealed inconsistent findings (Wu et al., 2024b). Regardless of the presence of depressive symptoms, elderly female participants with the WW classification significantly outperformed the IA group in measures of global cognitive performance. Furthermore, after adjusting for confounding factors, the WW pattern demonstrated a significant positive correlation with composite CF scores; notably, its effect size (β = 0.451, 95% CI [0.216, 0.685]) was numerically superior to that of the RE pattern (β = 0.153, 95% CI [0.085, 0.221]) (Wu et al., 2024b).

**Figure 2:**
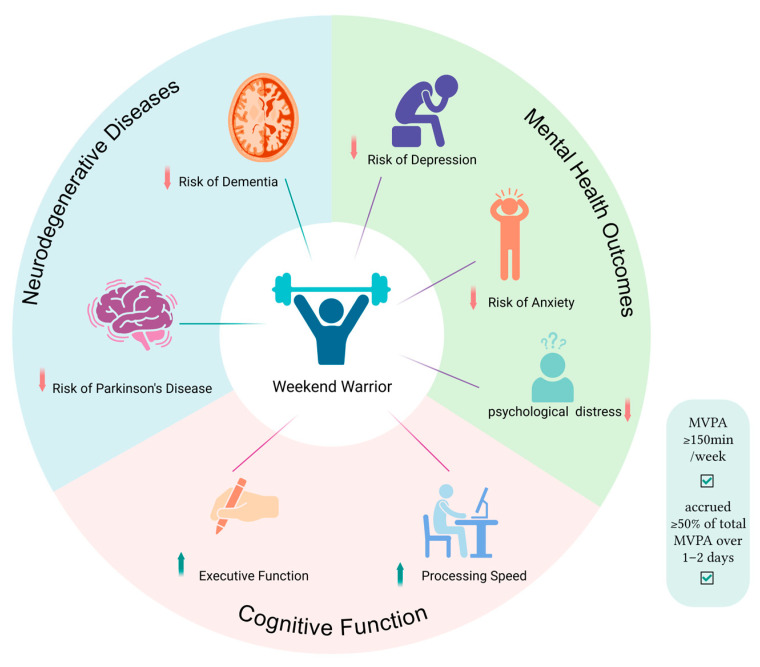
Summary of the association between the WW pattern and neuropsychological outcomes: The WW pattern (accumulating 150 min per week of MVPA over 1–2 days) is associated with beneficial outcomes across three domains. The blue and green sectors illustrate an association with lower risks for neurodegenerative diseases and mental health disorders, respectively. The pink sector illustrates improvements in CF such as processing speed and executive function.

A synthesis of all the aforementioned relevant results is visually presented in Figure 2.

## 4. Discussion

This systematic review provides a holistic synthesis of the extant evidence regarding the association of the WW pattern with neurodegenerative diseases—specifically dementia and Parkinson’s disease—as well as mental health conditions, including anxiety and depression. Overall, the analysis indicates that the WW pattern and RE share a similar relationship with favorable outcomes regarding these conditions and CF. Although heterogeneity in results was observed across specific subpopulations or study designs, the preponderance of evidence supports the conclusion that accumulating at least 50% of the recommended total MVPA volume within 1–2 days per week is adequate to be associated with a lower risk of these diseases and better cognitive outcomes. Our systematic analysis elucidates the substantial salutary benefits inherent in the WW pattern, thereby refining current insight into how such an intermittent form of PA modulates susceptibility to neuropsychological disorders and CF. The observed reductions in disease risk associated with the WW pattern suggest that individuals unable to adhere to a RE regime remain capable of eliciting robust salutary effects via condensed bouts of MVPA (Brawner & Keteyian, 2025).

Regarding the amelioration of neurodegenerative diseases, all included studies consistently indicated that the WW pattern was significantly associated with a lower risk of dementia and Parkinson’s disease. This suggests that PA patterns may be associated with substantial neurological benefits even when PA is concentrated rather than evenly distributed (Min et al., 2024; Ning et al., 2024, 2025; O’Donovan et al., 2025). Furthermore, current research on the WW pattern has encompassed populations from diverse geographical regions (O’Donovan et al., 2024; Xiao et al., 2018; Zhao et al., 2020) and included specific subgroups such as sedentary individuals and rural adults (Ning et al., 2025; Xiao et al., 2018). This supports the generalizability and positive associations of the WW pattern across varying regional, socioeconomic, and cultural contexts. However, Ning et al. (2024) noted that when a high volume of exercise (e.g., >300 min of MVPA per week) is compressed into a very short timeframe (specifically, >75% accumulated over 1–2 consecutive days), the previously observed potential associations between the WW pattern and dementia and Parkinson’s disease were no longer statistically significant. This suggests a potential dose-dependent limit to the neuroprotective effects of the WW pattern. However, when excessive PA is performed within a condensed timeframe, the WW pattern may not confer neurological benefits comparable to those of RE.

Regarding the amelioration of depression, the majority of included studies indicate that the WW pattern is significantly associated with lower depression risk (R. Chen et al., 2023; Liang et al., 2023; Liu et al., 2025; S. Xu et al., 2025), a consistent association across varying genetic risk profiles (Liu et al., 2025). Particularly among sedentary populations, the WW pattern appeared to yield even more pronounced improvements in depression risk compared to RE. This suggests that achieving the recommended volume of MVPA is the critical factor in mitigating depression risk—provided the total PA volume is met, compressing exercise into fewer sessions appears to confer comparable health benefits (R. Chen et al., 2023), which is consistent with previous research on the WW pattern indicating a negative correlation between PA participation and the severity of depression (Goodwin, 2003).

However, results from a study conducted in a Brazilian population presented a divergence: the WW pattern showed no significant association with the prevalence of depressive symptoms (de Victo et al., 2025), whereas RE consistently exhibited a significant association with the outcome. Furthermore, previous research has suggested that low-to-moderate intensity exercise may yield superior benefits for depression compared to high-intensity exercise (Rethorst et al., 2009). The observed discrepancies may stem from variations in study design and methodology, including differing definitions of WW (e.g., defined strictly as exercising 1–2 times per week), methods of PA assessment (e.g., International Physical Activity Questionnaire [IPAQ] or other self-report scales), participant characteristics (e.g., diagnosed clinical depression or depressive symptoms), and insufficient statistical power due to relatively small WW sample sizes. Moreover, the lack of control for social context (e.g., solitary or group exercise)—which plays a significant role in the association between PA and depressive symptoms (Kanamori et al., 2018)—may have limited the detection of the true effect of the WW pattern on depression. Despite these discrepancies, the synthesis of present evidence suggests that engagement in PA, regardless of the temporal distribution of exercise sessions, is associated with a reduction in depression risk (Schuch et al., 2018; S. Xu et al., 2025).

With respect to anxiety, the WW pattern conferred a statistically significant protective benefit. Notably, it confers benefits to individuals characterized by prolonged sedentary behavior and insufficient daily PA, while exhibiting a potential association comparable to that of RE across varying strata of genetic risk (Liu et al., 2025). Intriguingly, stratified analyses suggest that the beneficial effects of the WW pattern may be modulated by socioeconomic status. The association with a lower risk was particularly pronounced among low-income populations and patients with diabetes, whereas the impact appeared less distinct within high-income groups (Z. Chen et al., 2025); this implies that the WW pattern may represent a highly cost-effective intervention strategy for individuals with limited socioeconomic resources or concurrent chronic conditions. Nevertheless, it is important to acknowledge that variables such as per capita income and dietary habits may introduce residual confounding. Consistently, even when leisure-time PA volume fell below the minimum weekly recommendations, it remained associated with a lower risk of anxiety (Z. Chen et al., 2025; Min et al., 2024).

Our review further indicates that PA volume—whether achieved through the RE or WW pattern—is inversely correlated with psychological distress (Hamer et al., 2017). The observation that benefits peak when standard quotas (150 min per week of moderate or 75 min per week of vigorous exertion) are fulfilled underscores the paramount importance of total weekly volume over the specific frequency of sessions. Among individuals with chronic conditions, a reduced likelihood of psychological distress was observed even at PA levels below the recommended threshold. This observation may be attributable to the distinct therapeutic potential of low-to-moderate intensity PA in enhancing mood states (Moses et al., 1989) and alleviating fatigue symptoms (Puetz et al., 2006, 2008). Additionally, for certain subpopulations, high-intensity exercise may prove excessively challenging, potentially leading to lower adherence or discontinuation, thereby diminishing the opportunity to accrue mental health benefits (Ekkekakis, 2003; Ekkekakis et al., 2016).

Moreover, given that depression accelerates functional decline and is strongly correlated with cognitive deterioration—thereby significantly elevating the risk of dementia (Austin et al., 1999; Rashidi-Ranjbar et al., 2020)—we specifically investigated the interplay between the WW pattern and CF indices. In general, among the overall older adult population (not stratified by sex), RE was typically associated with improvements across a broader spectrum of cognitive domains—including memory, executive function, and processing speed—suggesting that consistent, long-term engagement is more conducive to building cognitive reserve (Wu et al., 2024a). In comparison, while the WW pattern also enhanced multiple CFs, its scope of impact appeared to be relatively more limited (Garcia-Estela et al., 2024; Wu et al., 2024a). This divergence in associated cognitive outcomes appears to be contingent upon participants’ mental health status and demographic characteristics. Notably, it was particularly pronounced among elderly individuals exhibiting depressive symptomatology, where only RE demonstrated a positive correlation with cognitive enhancement (Wu et al., 2024a). This finding aligns with the hypothesis that consistent PA participation over time might potentiate the fortification of cognitive reserve and the augmentation of neural plasticity (Colcombe et al., 2006). In contrast, among older women, the WW pattern was found to significantly improve cognitive performance regardless of the presence of depressive symptoms (Wu et al., 2024b).

While the observational nature of the included studies precludes the establishment of direct biological causality, synthesis with existing animal models and neurobiological evidence corroborates that depression is intrinsically linked to an imbalance between oxidative (pro-oxidant) and antioxidant systems, as well as structural and functional alterations in the hippocampus (Sarandol et al., 2007; Tartt et al., 2022). Consequently, the potential association between the WW pattern and depression may be related to its association with a lower risk through modulation of immune-inflammatory pathways. Specifically, this pattern has been shown to significantly suppress levels of tumor necrosis factor-α (TNF-α) and interleukin-6 (IL-6), effects which are directly associated with decreased neutrophil infiltration and the inhibition of lipid peroxidation (Öztürk et al., 2023). By mitigating the release of pro-inflammatory cytokines, these findings suggest that the WW pattern may ameliorate depressive symptoms by regulating inflammatory signaling pathways and attenuating neuroinflammation (Rakshe et al., 2024). In addition, the protective influence of the WW pattern on CF and neurodegenerative damage may be mediated through several mechanisms, including improved cerebral hemodynamics, neurogenesis (e.g., neuronal regeneration in the hippocampus) (La Tour et al., 2024; Li et al., 2024), the upregulation of relevant genes (Kandola et al., 2019; Keiser et al., 2024), and the alleviation of neuroinflammation (Rakshe et al., 2024).

**Strengths and limitations:** This systematic review integrates the WHO guidelines on PA and represents the first comprehensive evaluation of the association of the WW pattern with neurodegenerative diseases, mental health conditions, and CF. The primary strength of this study lies in its novelty: it is the first review to focus specifically on the health-promoting effects of the WW pattern within the neuropsychological domain, filling a critical gap left by previous research, which has predominantly concentrated on cardiovascular outcomes or all-cause mortality. Second, this review provides a holistic comparative assessment of the neuropsychological benefits associated with the WW pattern versus RE, offering fresh perspectives for public health recommendations.

However, these findings should be contextualized within the scope of certain study limitations. First, as the primary studies did not stratify for specific subpopulations, we cannot ascertain the suitability of this exercise protocol for special groups (e.g., individuals with musculoskeletal disabilities); therefore, future research must address populations with specific exercise needs. Additionally, the included literature consists exclusively of cohort and cross-sectional studies, lacking support from Randomized Controlled Trials (RCTs). This constrains our ability to definitively judge the stability of intervention effects and establish causal mechanisms. Crucially, while these included observational studies generally exhibited robust methodological designs, a significant limitation of the current literature lies in its overall strength of evidence, which is fundamentally constrained by measurement biases. The vast majority of the reviewed studies relied on subjective, self-reported questionnaires to assess PA. This reliance introduces substantial recall and social desirability biases, leading to potential misclassification of participants’ true activity status—a problem that is particularly acute when participants are asked to recall discrete, concentrated bouts of exercise like the WW pattern. Consequently, we must emphasize that high methodological quality scores in this context do not equate to a high strength of evidence. Future studies must transition towards objective quantification to establish the true impact of these specific PA patterns.

Furthermore, a critical methodological limitation of the current literature is the predominant reliance on indirect comparisons. Although our synthesis indicates that the WW and RE patterns generally yield comparable neuropsychological benefits, it is imperative to acknowledge that 12 of the 13 included studies exclusively utilized the IA population as the reference group. Consequently, the comparability between the WW and RE patterns was largely inferred by juxtaposing their respective effect sizes (e.g., HRs or ORs) against the IA baseline, rather than through direct head-to-head statistical testing. Given that indirect comparisons can be susceptible to unmeasured confounding across different cohorts, future observational studies and randomized controlled trials (RCTs) must explicitly establish the RE group as a reference category to rigorously quantify any true efficacy differences between the WW pattern and traditional regular exercise. Another critical limitation is the potential for reverse causality. Individuals experiencing early-stage cognitive decline or undiagnosed depressive symptoms may naturally reduce their PA levels, particularly intense weekend bouts, skewing the observed associations. Furthermore, despite most studies adjusting for various covariates, residual confounding remains a significant concern. Unmeasured or poorly quantified factors, such as unrecorded sleep quality over the weekend, dietary changes specific to rest days, and detailed baseline psychological resilience, could still confound the observed relationships.

Secondly, mechanistic research regarding the WW pattern remains largely confined to animal models, with a paucity of neurobiological studies in human samples. Consequently, relevant mechanisms have yet to be directly validated in clinical trials. Regarding psychological benefits, evidence suggests that, to isolate the impact of exercise patterns on mental health, it is crucial to control for exercise type and maintain consistency in training modalities to exclude the influence of confounding variables. Nevertheless, existing studies often failed to strictly control for variables such as exercise type, environmental setting, social interaction, and motivation levels, making it difficult to determine the independent contribution of the WW pattern to mental health improvements. Future experimental designs should employ standardized exercise protocols to exclude the impact of non-exercise factors on psychological metrics.

While this exercise pattern effectively accommodates work–life balance needs, potential drawbacks must be acknowledged; specifically, individuals engaging in intermittent, high-intensity exercise may be more susceptible to injury compared to those practicing RE with a more evenly distributed volume (Roberts et al., 2014).

As this review aimed to present a comprehensive overview, the included studies were predominantly based on large-scale databases from Western developed nations (e.g., UK Biobank and NHANES). Although data from Mexico and Brazil were included, there is a lack of direct evidence from indigenous cohorts in developing regions such as Asia and Africa. Given the significant disparities in lifestyle, socioeconomic status, and cultural backgrounds across countries, the health benefits of the WW pattern may vary among these populations. Moreover, demographic balance was not fully achieved, as the age range of included participants was broad and heavily skewed toward middle-aged and older adults, necessitating a cautious interpretation of the results.

**Implications:** The findings of this systematic review reflect an association between the WW pattern and enhanced psychological and cerebral health among adults, positioning it as a viable intervention strategy for augmenting CF in older populations. Given that the observed effects of the WW pattern mirror those of RE, public health initiatives should support involvement in all forms of PA; even sporadic accumulation of exercise can serve as an effective modality for promoting neuropsychological well-being. This offers an equipotent alternative strategy for individuals constrained by time who are otherwise unable to adhere to the standard frequency of MVPA recommended by the WHO.

## 5. Conclusions

After analyzing the included studies, it was concluded that the WW pattern offers considerable potential in mitigating the risk of neurodegenerative diseases, mental health conditions, as well as enhancing CF among adults. Crucially, this exercise pattern demonstrates potential protective associations of similar magnitude to those observed with RE although the evidence remains limited and heterogeneous.

This suggests that the total accumulated volume of PA may be a more critical determinant of neuropsychological health than the frequency of exercise sessions. These findings hold substantial public health implications: for individuals with time-constrained schedules, condensing the recommended exercise volume into 1–2 days offers a flexible and time-efficient alternative that still yields substantial benefits for cerebral and mental health. However, it must be noted that this conclusion relies predominantly on indirect comparisons against inactive populations, highlighting the need for future direct comparative studies. Future research should prioritize RCTs to validate these findings and definitively establish causal relationships. Moreover, in-depth exploration of the dose–response relationship of WW interventions is warranted. To substantiate the therapeutic potential of the WW pattern, future studies should leverage advanced neuroimaging techniques (e.g., functional magnetic resonance imaging [fMRI]) to elucidate the underlying biological mechanisms, specifically regarding neuroplasticity and neurotrophic factor expression.

## Figures and Tables

**Table 1:** Basic characteristics of included studies.

Author	Data Source	Study Type	Sample Size (%)	Group Age in Years (Mean ± SD or Median [IQR])	Follow Up (Years)	Males (%)	Baseline Age (Years)	Age Range (Years)	Main Outcomes	Covariates Included in the Adjustment Models Comprised
R. Chen et al. (2023)	NHANES 2007–2018	Cross-sectional study	Total: 21,125 Inactive: 10,602 (44.18%) Regular: 7002 (37.68%) Insufficiently active: 3124 (16.23%) WW: 397 (1.91%)	NA	NA	51.23	≥20	≥20	Participants classified as WW exhibited a decreased likelihood of depression when contrasted with IA populations. Moreover, comparisons revealed no notable disparities in risk reduction between WW cohort and those engaging in RE exercise.	Age, sex, ethnicity, educational attainment, income, marital status, BMI, smoking and drinking habits, history of CVD, diabetes, and hypertension.
Z. Chen et al. (2025)	NHANES 2007–2012	Cross-sectional study	Total: 13,740 Inactive: 7289 (46.08%) Regular: 3807 (32.24%) Insufficiently active: 2037 (16.53%) WW: 607 (5.15%)	No anxiety: 47.76 (17.17) Anxiety: 44.98 (15.32)	NA	48.84	47.04 averages	20–80	The WW pattern, like RE, is also significantly associated with a lower risk of anxiety, with this potential association being particularly pronounced among low-income populations and individuals with diabetes.	Age, sex, ethnicity, educational attainment, marital status, PIR, and BMI, lifestyle factors (smoking, alcohol consumption, sleep duration) and chronic comorbidities (hypertension, diabetes).
de Victo et al. (2025)	Brazil Hospital	Cross-sectional study	Total: 29,907 Not meeting PA recommendation: 12,861 (43.0%) Regular: 16,317 (54.6%) WW: 729 (2.4%)	Absence depressive symptoms: 44.72 (9.45) Presence depressive symptoms: 42.52 (9.82)	NA	71.20	44.48 averages	18–65	While RE engagement conferred a robust protective benefit, the WW pattern appeared to lack a statistically significant linkage to the development of depressive symptoms.	Age, sex, BMI, duration of sedentary behavior, tobacco and alcohol usage, self-perceived stress, glycated hemoglobin, total cholesterol, and history of conditions (hypertension, diabetes, dyslipidemia, lower urinary tract symptoms).
Hamer et al. (2017)	HSE and SHS	Cross-sectional study	Total: 108,011 Inactive: 54,655 (50.6%) Regular: 18,976 (17.57%) Insufficiently active: 25,459 (23.57%) WW: 8921 (8.26%)	Non-psychological distress: 46.9 (17.2) Psychological distress: 45.8 (16.6)	NA	46.50	47.00 averages	≥30	Relative to the IA baseline, even participants falling into the “insufficient activity” category demonstrated a reduced probability of psychological distress. Furthermore, both RE and WW patterns exhibited a robust inverse association with psychological distress.	Age, sex, smoking behavior, socio-occupational classification, BMI, longstanding illness, survey year.
Liang et al. (2023)	NHANES 2007–2020	Cross-sectional study	Total: 23,258 Inactive: 9781 (42.05%) Regular: 6366 (27.37%) Insufficiently active: 6069 (26.09%) WW: 1042 (4.48%)	Inactive: 45.89 (0.38) Regular: 41.98 (0.34) Insufficiently active: 47.87 (0.36) WW: 47.46 (0.81)	NA	52.30	45.49 averages	≥20	Relative to the IA cohort, both the WW and regularly active groups exhibited an inverse association with depressive symptomatology; furthermore, a linear dose–response relationship was identified linking the aggregate volume of weekly PA to the risk of depression	Age, sex, ethnicity, educational attainment, marital status, PIR, BMI, lifestyle variables (smoking status, alcohol consumption, duration of sedentary behavior, and sleep duration).
Liu et al. (2025)	UK Biobank	Prospective cohort	Total: 84,570 Inactive: 27,645 (32.7%) Regular: 20,300 (24.0%) WW: 36,625 (43.3%)	Inactive: 57.3 (7.8) Regular: 55.0 (7.8) WW: 56.1 (7.7)	9.4 years averages	45.50	56.20 averages	NA	Participants in the RE and WW categories enjoyed a substantial protective benefit against depression and anxiety compared to the IA baseline.	Sex, ethnicity, educational attainment, BMI, and the Townsend deprivation score, lifestyle factors (smoking status, frequency of alcohol intake, dietary patterns, sleep), clinical history (type 2 diabetes, CVD).
Min et al. (2024)	UK Biobank	Prospective cohort	Total: 75,629 Inactive: 24,365 (32.2%) Regular: 21,291 (28.2%) WW: 29,973 (39.6%)	NA	8.4 years averages	44.60	61.80 averages	NA	Similarly, the WW protocol shows a potential association with neurological and psychiatric outcomes comparable to that of RE engagement.	Age, sex, ethnicity, BMI, TDI, educational attainment, lifestyle factors (smoking status, alcohol intake frequency, diet scores and sleep scores), clinical history (diabetes, hypertension, cancer).
Ning et al. (2024)	UK Biobank	Prospective cohort	Total: 92,784 Inactive: 31,514 (33.97%) Regular: 21,053 (22.69%) WW: 40,217 (43.34%)	Inactive:64.0 [57.0–69.0] Regular: 61.0 [54.0–67.0] WW: 63.0 [56.0–68.0]	8.8 years averages	43.62	61.88 averages	≥40	Participants in the WW pattern and those who follow the conventional MVPA pattern have a similar risk of developing NDD.	Age, sex, ethnicity, TDI, educational attainment, employment status, lifestyle factors (smoking, alcohol consumption status, diet quality), and comorbidities.
Ning et al. (2025)	UK Biobank	Prospective cohort	Total: 91,948 Inactive: 31,064 (33.78%) Regular: 34,366 (37.38%) WW: 26,518 (28.84%)	Average sedentary time: Inactive: ≥8.85 h/day 64.2 [56.8–69.2] <8.85 h/day 64.0 [57.0–68.6] Regular: ≥8.85 h/day 61.6 [54.4–67.3] <8.85 h/day 62.5 [55.7–67.4] WW: ≥8.85 h/day 62.6 [55.3–67.8] <8.85 h/day 63.1 [56.3–67.8]	7.9 years averages	43.50	63.00 averages	NA	In individuals who sit for long periods, concentrating the weekly recommended MVPA into 1–2 days is significantly associated with a reduced risk of dementia.	Age, sex, ethnicity, BMI, TDI, educational attainment, employment status, lifestyle factors (alcohol and cigarette use), and baseline comorbid conditions including cardiovascular disease, hypertension, and diabetes.
O’Donovan et al. (2025)	The Mexico City Prospective Study	Prospective cohort	Total: 10,033 no sport or exercise: 7945 (79.19%) Regular: 1362 (13.58%) WW: 726 (7.24%)	No sport or exercise: 51.1 (10.7) Regular: 52.2 (10.8) WW: 49.5 (10.5)	16.2 years averages	29.98	51.00 averages	≥35	Both WW and RE sports activity patterns are linked to a lower risk of mild dementia.	Age, sex, educational attainment, income, systolic blood pressure, smoking, BMI, civil status, sleep duration, fruit and vegetable intake, Alcohol drinking.
Wu et al. (2024a)	NHANES 2011–2014	Cross-sectional study	Total: 2877	Non-depressive symptoms: 69.18 (6.66) Depressive symptoms: 69.15 (6.62)	NA	54.90	69.17 averages	NA	While benefits to CF were confined to the RE group among depressed older adults, both WW and RE patterns proved beneficial for cognitive health in those free of depression.	Age, sex, education attainment, ratio of family income to poverty, alcohol frequency, waist circumference, BMI, smoking status, diabetes, sleep duration, sedentary time.
Wu et al. (2024b)	NHANES 2011–2014	Cross-sectional study	Total: 1507 Inactive: 814 (54.01%) Regular: 663 (43.99%) WW: 30 (1.99%)	Inactive: 70.36 (6.81) Regular: 68.34 (6.44) WW: 72.02 (6.38)	NA	0	70.24 averages	NA	A favorable correlation exists between physical exertion and cognitive performance in older women. Notably, condensing activity into shorter durations—characteristic of the Weekend Warrior mode—correlates equally with neurocognitive preservation.	Race, age, education attainment, ratio of family income to poverty, alcohol frequency, waist circumference, BMI, smoking status, diabetes, depressive symptom, sleep disorders.
S. Xu et al. (2025)	NHANES 2011–2014	Cross-sectional study	Total: 6080 Inactive: 141 (2.32%) Regular: 3192 (52.50%) Insufficiently active: 933 (15.35%) WW: 1814 (29.84%)	Inactive: 71.74 (11.54) Regular: 45.95 (17.24) Insufficiently active: 60.78 (16.72) WW: 46.98 (17.57)	NA	48.00	49.13 averages	18–80	While all active groups showed reduced depression risks compared to inactivity, the RE protocol generally yielded superior outcomes over the WW and IA groups.	Age, sex, ethnicity, BMI, education attainment, marital status, income level, smoking status, alcoholism, and sedentary time.

Notes: BMI Body Mass Index, CVD cardiovascular Disease, PIR Poverty Income Ratio, TDI Townsend Deprivation Index.

**Table 2:** WW characteristics of included studies.

Author	Country (First Author)	Main Physical Activity Assessment	Physical Activity Patterns	Questionnaire/Measurement Methods	Main Analytical Methods
R. Chen et al. (2023)	China	Self-reported	Inactive/Regular/Insufficient/WW	Patient Health Questionnaire-9 (PHQ-9)	Multivariate logistic regression
Z. Chen et al. (2025)	China	Self-reported	Inactive/Regular/Insufficient/WW	Global Physical Activity Questionnaire (GPAQ) Patient Health Questionnaire-9 (PHQ-9)	Multivariate logistic regression and weighted logistic regression
de Victo et al. (2025)	Brazil	Self-reported	Not meeting PA recommendation/Regular/WW	Physical Activity Questionnaire (IPAQ-LF) Beck Depression Inventory-II (BDI-II)	Binary logistic regression.
Hamer et al. (2017)	UK	Self-reported	Inactive/Regular/Insufficient/WW	12 item version of the General Health Questionnaire (GHQ-12)	Binary logistic regression.
Liang et al. (2023)	China	Self-reported	Inactive/Regular/Insufficient/WW	Patient Health Questionnaire-9 (PHQ-9)	Survey-multivariable logistic regression
Liu et al. (2025)	China	Accelerometry	Inactive/Regular/WW	Patient Health Questionnaire (PHQ)	Cox regression
Min et al. (2024)	China	Accelerometry	Inactive/Regular/WW	Composite brain health score	Cox proportional hazards regression
Ning et al. (2024)	China	Accelerometry	Inactive/Regular/WW	NA	Cox proportional hazards regression
Ning et al. (2025)	China	Self-reported	Inactive/Regular/WW	Medical Record (International Classification of Diseases,10th Revision, ICD-10)	Cox proportional hazards regression
O’Donovan et al. (2025)	Mexico	Self-reported	No sports or exercise/Regular/WW	The Mini-Mental State Examination (MMSE)	Cox proportional hazards regression
Wu et al. (2024a)	China	Self-reported	Inactive/Regular/WW	Global Physical Activity Questionnaire (GPAQ) Patient Health Questionnaire-9 (PHQ-9) The Consortium to Establish a Registry for Alzheimer’s Disease Word List (CERAD-WL) Animal Fluency (AF) test Digit Symbol Substitution Test (DSST)	Multivariate linear regression
Wu et al. (2024b)	China	Self-reported	Inactive/Regular/WW	Global Physical Activity Questionnaire (GPAQ) The Consortium to Establish a Registry for Alzheimer’s Disease Word List (CERAD-WL) Animal Fluency (AF) test Digit Symbol Substitution Test (DSST)	Multivariate linear regression
S. Xu et al. (2025)	China	Self-reported	Inactive/Regular/Insufficient/WW	Patient Health Questionnaire-9 (PHQ-9)	Multivariate logistic regression

**Table 3:** Operational definitions of Weekend Warrior across studies.

Author	Guideline-Based Threshold	Number of Days/Sessions	Days/Sessions Proportion Requirement	Exact Definition of WW
R. Chen et al. (2023)	≥150 min/week of moderate-intensity aerobic activity, or at least 75 min/week of vigorous-intensity aerobic activity, or equivalent combinations.	1–2 sessions/week	NA	Weekend warrior: at least 150 min of total PA in 1 or 2 sessions per week was reported.
Z. Chen et al. (2025)	≥150 min of moderate-intensity exercise or 75 min of vigorous-intensity exercise per week	1–2 sessions/week	NA	Those who met or exceeded 150 min of MVPA weekly were categorized as “weekend warriors.
de Victo et al. (2025)	≥150 min per week of MVPA or 75 min of VPA	1–2 sessions/week	NA	Weekend warriors for those who accumulated the weekly recommendation in one or two sessions
Hamer et al. (2017)	≥150 min MPA/week or 75 min VPA/week or a combination of the two, as well as at least 2 days of muscle strength exercise per week	1–2 sessions/week	NA	Weekend warrior was defined as reporting ≥150 min/week in moderate-intensity activities or ≥75 min/week in vigorous-intensity activities from one or two sessions.
Liang et al. (2023)	≥150 min of MPA or 75 min of VPA per week	1–2 sessions/week	NA	Weekend warrior, reporting at least 150 min/wk. in moderate-intensity or at least 75 min/week. in vigorous-intensity activities from 1 or 2 sessions.
Liu et al. (2025)	≥150 min MVPA/week	1–2 days/week	≥50%	MVPA ≥ 150 min/week and ≥50% of total MVPA over 1 to 2 days.
Min et al. (2024)	≥150 min MVPA/week	1–2 days/week	≥50%	Weekend warrior was defined as ≥150 min of MVPA per week and had ≥50% of total MVPA over 1–2 days.
Ning et al. (2024)	≥150 min of MPA or 75 min of VPA per week	1–2 sessions/week	≥50% or 75% of recommended MVPA	Weekend warrior, reporting at least 150 min/week. in moderate-intensity or at least 75 min/week. in vigorous-intensity activities from 1 or 2 sessions.
Ning et al. (2025)	≥150 min MVPA/week	1–2 days/week	≥50%	weekend warrior (WW), which is characterized by at least 150 min of MVPA concentrated within 1 to 2 days (mostly weekends) per week.
O’Donovan et al. (2025)	NA	1–2 sessions/week	NA	The ‘weekend warrior’ group included those who said they exercised or played sports up to once or twice per week.
Wu et al. (2024a)	≥150 min PA/week	1–2 sessions/week	NA	Individuals meeting or exceeding 150 min of total PA weekly within one or two sessions.
Wu et al. (2024b)	≥150 min PA/week (equating 1 min of VPA to 2 min of MPA)	1–2 sessions/week	NA	WW for individuals completing at least 150 min of PA in one or two sessions.
S. Xu et al. (2025)	≥150 min of MPA, or ≥75 min of VPA or an equivalent combination	1–2 days/week	≥50%	Weekend warrior (WW), which is characterized by at least 150 min of MVPA concentrated within 1 to 2 days (mostly weekends) per week.

Notes: MPA Moderate-intensity Physical Activity, VPA Vigorous-intensity Physical Activity.

**Table 4:** Quality evaluation of cohort studies.

Study	Year	Selection	Comparability	Outcome	Total
Cohort studies (*n* = 5)					
Liu et al. (2025)	2025	****	**	**	8
Min et al. (2024)	2024	****	**	**	8
Ning et al. (2024)	2024	****	**	***	9
Ning et al. (2025)	2025	****	**	**	8
O’Donovan et al. (2025)	2025	****	**	***	9

The NOS scale served as the tool for evaluating the quality of the cohort studies. Note: The number of asterisks denotes the quality rating: low (0–3 stars), moderate (4–6 stars), and high (7–9 stars).

**Table 5:** Quality evaluation of cross-sectional studies.

Study	Year	1	2	3	4	5	6	7	8	9	10	11	Total
Cross-sectional studies (*n* = 8)													
R. Chen et al. (2023)	2023	1	1	1	1	1	1	1	1	1	1	1	11
Z. Chen et al. (2025)	2025	1	1	1	1	1	1	1	1	1	1	1	11
de Victo et al. (2025)	2025	1	1	0	1	1	1	1	1	1	1	1	10
Hamer et al. (2017)	2017	1	1	1	1	1	1	1	1	1	1	1	11
Liang et al. (2023)	2023	1	1	1	1	1	1	1	1	1	1	1	11
Wu et al. (2024a)	2024	1	1	1	1	1	1	1	1	1	1	1	11
Wu et al. (2024b)	2024	1	1	1	1	1	1	1	1	1	1	1	11
S. Xu et al. (2025)	2025	1	1	1	1	1	1	1	1	1	1	1	11

The AHRQ scale served as the tool for evaluating the quality of the cross-sectional studies.

## Data Availability

No new data were created or analyzed in this study.
